# Evaluating NAT2PRED for inferring the individual acetylation status from unphased genotype data

**DOI:** 10.1186/1471-2350-10-148

**Published:** 2009-12-31

**Authors:** Audrey Sabbagh, Pierre Darlu, Michel Vidaud

**Affiliations:** 1 INSERM UMR745, Faculty of Pharmacy, University of Paris Descartes, Paris, France; 2 Department of Biochemistry and Molecular Genetics, Beaujon hospital, AP-HP, Clichy, France; 3 INSERM U535, Paul Brousse Hospital, Villejuif, France; 4 University of Paris-Sud, UMR-S535, Villejuif, France

## Abstract

**Background:**

Genetically determined differences in *N*-acetylation capacity have proved to be important determinants of both the effectiveness of therapeutic response and the development of adverse drug reactions and toxicity during drug treatment. NAT2PRED is a web-server that allows a fast determination of NAT2 acetylation phenotype from genotype data without taking the extra step of reconstructing haplotypes for each individual (publicly available at http://nat2pred.rit.albany.edu). However, the classification accuracy of NAT2PRED needs to be assessed before its application can be advocated at a large scale.

**Methods:**

The ability of NAT2PRED to classify individuals according to their acetylation status (slow, intermediate and rapid acetylators) was evaluated in a worldwide dataset composed of 56 population samples (8,489 individuals) from four continental regions.

**Results:**

NAT2PRED correctly identified slow acetylators with a sensitivity above 99% for all populations outside sub-Saharan Africa. Nevertheless, NAT2PRED showed a poor ability to distinguish between intermediate and rapid acetylators, with a classification error rate reaching up to 10% in the non-African samples.

**Conclusion:**

NAT2PRED is an excellent tool to infer the individual acetylation status from *NAT2 *genotype data when the main interest is to distinguish slow acetylators from the others. This should facilitate the determination of the individual acetylation status in routine clinical practice and lead to better monitoring of risks associated with cancer and adverse drug reactions.

## Background

The human acetylation polymorphism is one of the most intensively studied pharmacogenetic traits that underlie interindividual and interethnic differences in response to xenobiotics. Genetically determined differences in *N*-acetylation capacity have proved to be important determinants of both the effectiveness of therapeutic response and the development of adverse drug reactions and toxicity during drug treatment [1,2]. Some of the drugs excreted by acetylation are crucial in the treatment of diseases representing a worldwide concern, such as tuberculosis, AIDS-related complex diseases, and hypertension. Moreover, numerous association studies have linked the acetylation phenotype to susceptibility to a variety of complex human diseases, the most consistent findings being those regarding urinary bladder cancer, asthma and other allergic disorders [3-6].

Single nucleotide polymorphisms (SNPs) in the coding region of the *NAT2 *gene determine the acetylation phenotype. Thanks to the well-established genotype-phenotype correlation at this locus, the individual acetylation status can be reliably predicted from the haplotype combination at *NAT2*, according to the acknowledged classification of *NAT2 *haplotypes into either low-activity or fully functional alleles (see the consensus *NAT2 *gene nomenclature website: http://www.louisville.edu/medschool/pharmacology/NAT.html). Slow, intermediate and rapid acetylators are defined as carriers of zero, one or two functional haplotypes, respectively. Many studies did not distinguish between rapid and intermediate acetylators, categorizing both types of subjects as rapid acetylators.

Determining the linkage phase of SNP alleles along the coding sequence of *NAT2 *is crucial to unequivocally assign an individual's multi-site *NAT2 *genotype to a particular combination of two multilocus haplotypes and to correctly infer his acetylation status. Ambiguous *NAT2 *genotyping data that may lead to patient misclassification appear indeed to be common in human populations [7]. Special analytical techniques have been designed to unambiguously determine the allocation of SNP alleles to either DNA strand. However, molecular haplotyping methods are labour-intensive and expensive and are not suitable for routine clinical applications. A cheap and straightforward alternative for haplotype reconstruction is the use of computational algorithms. Several studies have demonstrated the high effectiveness of haplotype reconstruction algorithms in the particular case of the *NAT2 *gene by comparing the computationally inferred haplotypes to the real ones resolved through the use of molecular haplotyping techniques [7-9]. But such *in silico *approaches may not be convenient to clinicians not familiar with computational haplotype analysis. Moreover, algorithmic techniques are statistical and require the analysis of a population rather than a single or a few individuals.

To circumvent these limitations, Kuznetsov *et al. *[10] have developed a web-server NAT2PRED that allows a fast determination of NAT2 acetylation phenotypes (slow, intermediate, and rapid) from the unphased genotype data at six polymorphic positions in *NAT2*. These six SNPs are the most commonly reported ones in surveys of *NAT2 *sequence variation in human populations [11]. NAT2PRED alleviates the need of reconstructing haplotypes by implementing a supervised pattern recognition method that was trained on the *NAT2 *genotyping data of 1,377 subjects of known acetylation status. This tool is publicly available http://nat2pred.rit.albany.edu and has a simple intuitive user interface. It showed a nearly perfect classification accuracy of 99.9% in a sample mostly composed of Caucasians [10]. However, it remains unclear to what extent this tool can be applied to individuals from any ethnicity since it was developed on a dataset where 94% of subjects were Caucasian. The accuracy of NAT2PRED needs to be assessed before its application can be advocated at a large scale.

The objective of the present study was to empirically evaluate the performance of NAT2PRED in a wide collection of population samples worldwide. To this end, we performed an extensive survey of the literature to identify those samples that were adequately genotyped for all the common SNPs in *NAT2*. In total the collected data consisted of 8,489 individuals from 56 human populations representing major geographic regions: sub-Saharan Africa (12 samples), Europe and North Africa (23), Central/South Asia (5), East Asia (13), and America (3). In each sample, *NAT2 *haplotypes were reconstructed using either molecular or computational methods. Therefore, these data provided an opportunity to compare, for each subject, the acetylation status inferred by NAT2PRED from the unphased *NAT2 *genotype data with the "true" one predicted from the pair of haplotypes reconstructed through molecular or computational haplotyping. NAT2PRED correctly identified slow acetylators with a sensitivity above 99% for all populations outside sub-Saharan Africa.

## Methods

We selected from published reports up to November 2009 all the population samples that were genotyped for the seven most common SNPs in *NAT2 *(191G>A (rs1801279), 282C>T (rs1041983, rs59855457), 341T>C (rs1801280, rs56935242), 481C>T (rs1799929, rs60310310), 590G>A (rs1799930, rs60190029), 803A>G (rs1208, rs56599719, rs58999469), and 857G>A (rs1799931, rs58803786)) and for which genotype frequency data were available in the paper. We also included those non-African samples that were genotyped for all SNPs except SNP 191G>A as this is rare in non-African populations. The final dataset used for the present study describe 56 population samples from throughout the world, representing 8,489 individuals. A summary description of the data set is provided in Additional File 1.

In each sample, *NAT2 *haplotypes were either directly resolved using molecular-haplotyping techniques (through allele-specific PCR and restriction mapping) or were computationally inferred from the unphased multilocus genotypes using statistical algorithms (based either on a parsimony, maximum-likelihood, or Bayesian approach). For some samples, a combination of the two approaches was used. The acetylation phenotype of each individual was then predicted by assuming that the homozygous or compound heterozygous genotype for two haplotypes of the series NAT2*4, NAT2*11, NAT2*12 or NAT2*13 results in the rapid acetylator status, the occurrence of one of these haplotypes in combination with a haplotype of the series NAT2*5, NAT2*6, NAT2*7 or NAT2*14 results in the intermediate acetylator status and the occurrence of two haplotypes of the series NAT2*5, NAT2*6, NAT2*7 or NAT2*14 results in the slow acetylator phenotype.

Besides, NAT2PRED was applied to the unphased genotype data of each sample (at the six polymorphic positions 282, 341, 481, 590, 803, and 857) to provide an inferred acetylation status that was compared to the "true" one predicted from the diplotype configuration at *NAT2*. The web-server was used in the standard way, by specifying the genotype at each polymorphic position using radio buttons and clicking the 'Submit' button. We retained for each subject the final prediction (i.e., the phenotype with the highest probability). The performance of NAT2PRED was measured by computing in each sample the classification error rate, which is the proportion of individuals whose acetylation status was incorrectly inferred by the web-server. The classification error rate of NAT2PRED was evaluated by considering either two (slow vs. others) or three (slow vs. intermediate vs. rapid) phenotypic classes for the acetylation status.

## Results and discussion

**Figure 1:**
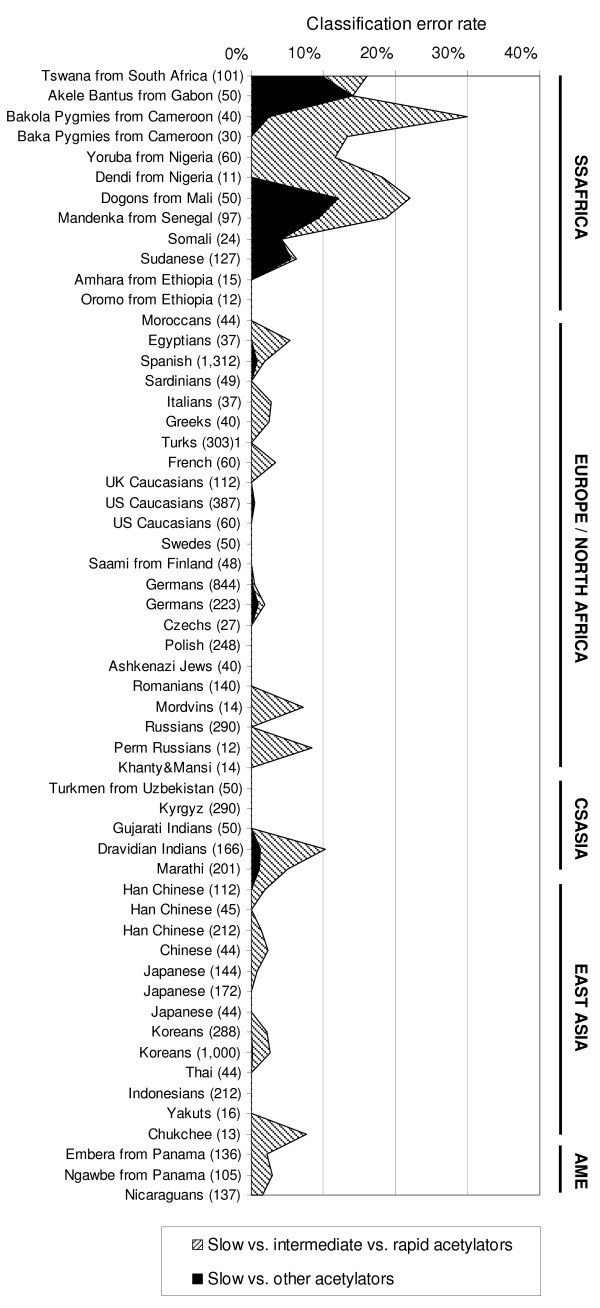
**Classification error rates of NAT2PRED in the 56 worldwide samples**. The classification accuracy of NAT2PRED was evaluated using either three phenotypic classes (slow, intermediate and rapid acetylators; hatched area) or two phenotypic classes (slow and other acetylators; black area) for the acetylation status. Single populations are reported on the left side of the plot, with sample sizes (number of individuals) in brackets. Geographic areas are indicated on the right side, as follows: SSAFRICA, sub-Saharan Africa; EUROPE/NORTH AFRICA, Europe and North Africa; CSASIA, Central and South Asia; EAST ASIA, East Asia; AME, America. The assignment of populations to one of the five world regions was based on the origin of the population, in effect ignoring the past 1,000 years of known human migration (e.g., people of European descent in the United States were assigned to Europe).

The classification error rates of NAT2PRED in each sample for both classification issues (two or three acetylation phenotypes) are shown in Figure 1 (see Additional File 1 for the exact numbers). The performance of this tool in each of the five world regions investigated is summarized in Table 1. NAT2PRED performed poorly in almost all sub-Saharan African samples investigated, except in the two Ethiopian samples where it correctly classified 100% of subjects into rapid, intermediate and slow acetylators. Such a poor performance is not surprising in view of the omission by the supervised learning classifier implemented in NAT2PRED of the functional SNP 191G>A which, although monomorphic in most worldwide populations, occurs at an appreciable frequency in many sub-Saharan African populations (up to 23%) and accounts for a sizeable portion of the slow acetylators encountered in these populations [11,12]. By contrast, NAT2PRED achieved a high classification accuracy in all the other world regions. In particular, individuals with a slow acetylation phenotype were correctly identified as slow acetylators with a sensitivity above 99% for all populations outside sub-Saharan Africa. Conversely, NAT2PRED showed a poor ability to distinguish between intermediate and rapid acetylators, with a classification error rate reaching up to 10% in the non-African samples.

**Table 1:** Classification error rates of NAT2PRED in each geographic region^a^.

	Classification error rate (%)	
	
	Two acetylation phenotypes ^b^	Three acetylation phenotypes ^c^
Sub-Saharan Africa (*n *= 617)	6.81% [0-14%]	14.26% [0-30%]
Europe and North Africa (*n *= 4,391)	0.32% [0-1%]	0.98% [0-8%]
Central/South Asia (*n *= 757)	0.53% [0-1%]	3.57% [0-10%]
East Asia (*n *= 2,346)	0.09% [0-0.2%]	1.71% [0-8%]
America (*n *= 378)	0%	2.12% [0-3%]

Values are the classification error rates of NAT2PRED when all population samples within a geographic area were pooled into a single sample. The range of variation of the classification error rate across individual samples is indicated in square brackets.

^a^See Figure 1 for the population composition of each geographic area.

^b^Classification error rate when two phenotypic classes are considered: slow vs. other acetylators (intermediate and rapid acetylators pooled together).

^c^Classification error rate when three phenotypic classes are considered: slow, intermediate and rapid acetylators.

It is important to note that for most of the samples investigated, *NAT2 *haplotypes were not actually determined by a molecular resolution of mutation linkage patterns but were rather inferred through computational methods (see Additional File 1). As real *NAT2 *haplotypes may be quite different from those calculated by computational inference [7], it is of high relevance to evaluate the performance of NAT2PRED in only those samples where the real *NAT2 *haplotypes were determined. There are only six population samples where molecular techniques were used to reconstruct haplotypes and applied to the whole set of individuals. In the single African sample (Tswana from South Africa), NAT2PRED showed a poor classification accuracy with a classification error rate of 10% and 16% when considering two (slow vs. others) or three (slow vs. intermediate vs. rapid) phenotypic classes for the acetylation status, respectively. In the five remaining non-African samples, the ability of NAT2PRED to distinguish slow acetylators from the others was very high with an average classification error rate of only 0.5%, ranging from 0% in UK Caucasians and Nicaraguans to 1.2% in Dravidian Indians. This means that in 99.5% of cases, NAT2PRED correctly identified slow acetylators in the same way the usual procedure did (that is, by reconstructing the real haplotypes through molecular techniques and by inferring the individual acetylation status from the haplotype combination at *NAT2*). On the other hand, a poorer performance was observed when intermediate acetylators were to be distinguished from rapid acetylators in these five samples (average error rate of 2.6%, ranging from 0% to 10.2%).

## Conclusion

Our results show that NAT2PRED is an excellent tool to infer the individual acetylation status from *NAT2 *genotype data when the main interest is to distinguish slow acetylators from the others. Its classification error rate is indeed too high to ensure a reliable discrimination between intermediate and rapid acetylators. However, since the slow acetylator phenotype is the one which exhibits today the strongest association with susceptibility to bladder cancer and adverse drug reactions [2,3], the web-server NAT2PRED should be of great use in clinical laboratories. This should facilitate the determination of the individual acetylation status in routine clinical practice and lead to better monitoring of risks associated with cancer and adverse drug reactions. On the other hand, NAT2PRED must be used with caution for patients whose population of origin contains a sizeable frequency of SNP 191G>A. This concerns populations of African origin but also several non-African populations, notably South-European and South-American populations, where this SNP has been identified, probably as a consequence of genetic admixture with Africans [13]. In particular, a frequency above 1% has been reported for the 191A allele in Spanish (1.4%) [7], in Coyaima from Colombia (3.5%) [14], in Brazilians from Rio de Janeiro (4.7%) [15], and in Brazilians from Goiàs (2.4%) [15].

## Competing interests

The authors declare that they have no competing interests.

## Authors' contributions

AS: planned the analysis, collected and analyzed the data, drafted the manuscript. PD and MV: participated in the interpretation of data and critically revised the manuscript. All authors have read and approved the content of the manuscript.

## Pre-publication history

The pre-publication history for this paper can be accessed here:

http://www.biomedcentral.com/1471-2350/10/148/prepub

## Supplementary Material

Additional file 1 **Table S1**. Classification error rates of NAT2PRED in the 56 worldwide samples collected from the literature. Click here for file
