# Supplementary material for: Evaluating NAT2PRED for inferring the individual acetylation status from unphased genotype data
Source: BMC Med Genet. 2009 Dec 31;10:148. doi: 10.1186/1471-2350-10-148 (PMC2806877; doi:10.1186/1471-2350-10-148)
Supplement: Additional file 1 — Table S1. Classification error rates of NAT2PRED in the 56 worldwide samples collected from the literature. [file 1471-2350-10-148-S1.DOC]

**Table S1: Classification error rates of NAT2PRED in the 56 worldwide samples collected from the literature.**

**Table S1 (Continued)**

^a^ All population samples were genotyped for the seven common SNPs of the *NAT2* gene (191G>A, 282C>T, 341T>C, 481C>T, 590G>A, 803A>G, and 857G>A), except some non-African samples where the SNP 191G>A was omitted since this is monomorphic in non-African populations.

^b^ In each sample, *NAT2* haplotypes were reconstructed using either molecular techniques (M), computational algorithms (C), or a combination of both approaches (M+C; some studies indeed limited the application of molecular haplotyping to particular cases as those where an alternative linkage pattern of mutations would have led to a change in phenotype). C_B_, C_E_ and C_C_ refer to the computational method used (when applicable): the Bayesian algorithm implemented in PHASE^26^, the EM algorithm^27^ implemented in Haploview^28^, and the Clark’s method^29^ based on maximum parsimony, respectively.

^c^ Classification error rate of NAT2PRED when two phenotypic classes were considered: slow and other acetylators (intermediate and rapid acetylators pooled together).

^d^ Classification error rate of NAT2PRED when three phenotypic classes were considered: slow, intermediate and rapid acetylators.

**References**

1. Loktionov A, Moore W, Spencer SP, Vorster H, Nell T, O'Neill IK, Bingham SA, Cummings JH. Differences in N-acetylation genotypes between Caucasians and Black South Africans: implications for cancer prevention. *Cancer Detect. Prev.* **26**, 15-22 (2002).

2. Patin E, Barreiro LB, Sabeti PC, Austerlitz F, Luca F, Sajantila A, Behar DM, Semino O, Sakuntabhai A, Guiso N, Gicquel B, McElreavey K, Harding RM, Heyer E, Quintana-Murci L: Deciphering the ancient and complex evolutionary history of human arylamine N-acetyltransferase genes. *Am. J. Hum. Genet.* **78**, 423-436 (2006).

3. The International HapMap Consortium. The International HapMap Project. *Nature* **426**, 789-796 (2003).

4. Luca F, Bubba G, Basile M, Brdicka R, Michalodimitrakis E, Rickards O, Vershubsky G, Quintana-Murci L, Kozlov AI, Novelletto A. Multiple advantageous amino acid variants in the NAT2 gene in human populations. *PLoS ONE* **3**, e3136 (2008).

5. Delomenie C, Sica L, Grant DM, Krishnamoorthy R, Dupret JM. Genotyping of the polymorphic N-acetyltransferase (NAT2*) gene locus in two native African populations. *Pharmacogenetics* **6**, 177-185 (1996).

6. Sabbagh A, Langaney A, Darlu P, Gérard N, Krishnamoorthy R, Poloni ES. Worldwide distribution of NAT2 diversity: implications for NAT2 evolutionary history. *BMC Genet.* **9**, 21 (2008).

7. Al-Yahyaee S, Gaffar U, Al-Ameri MM, Qureshi M, Zadjali F, Ali BH, Bayoumi R. N-acetyltransferase polymorphism among northern Sudanese. *Hum. Biol.* **79**, 445-452 (2007).

8. Agúndez JA, Golka K, Martínez C, Selinski S, Blaszkewicz M, García-Martín E. Unraveling ambiguous NAT2 genotyping data. *Clin. Chem.* **54**, 1390-1394 (2008).

9. Aynacioglu AS, Cascorbi I, Mrozikiewicz PM, Roots I. Arylamine N-acetyltransferase (NAT2) genotypes in a Turkish population. *Pharmacogenetics* **7**, 327-331 (1997).

10. Deitz AC, Zheng W, Leff MA, Gross M, Wen WQ, Doll MA, Xiao GH, Folsom AR, Hein DW. N-Acetyltransferase-2 genetic polymorphism, well-done meat intake, and breast cancer risk among postmenopausal women. *Cancer Epidemiol. Biomarkers Prev.* **9**, 905-910 (2000).

11. Cascorbi I, Drakoulis N, Brockmoller J, Maurer A, Sperling K, Roots I. Arylamine N-acetyltransferase (NAT2) mutations and their allelic linkage in unrelated Caucasian individuals: correlation with phenotypic activity. *Am. J. Hum. Genet.* **57**, 581-592 (1995).

12. Schnakenberg E, Lustig M, Breuer R, Werdin R, Hubotter R, Dreikorn K, Schloot W. Gender-specific effects of NAT2 and GSTM1 in bladder cancer. *Clin. Genet.* **57**, 270-277 (2000).

13. Mrozikiewicz PM, Cascorbi I, Brockmoller J, Roots I. Determination and allelic allocation of seven nucleotide transitions within the arylamine N-acetyltransferase gene in the Polish population. *Clin. Pharmacol. Ther.* **59**, 376-382 (1996).

14. Rabstein S, Unfried K, Ranft U, Illig T, Kolz M, Rihs HP, Mambetova C, Vlad M, Bruning T, Pesch B. Variation of the N-acetyltransferase 2 gene in a Romanian and a Kyrgyz population. *Cancer Epidemiol. Biomarkers Prev.* **15**, 138-141 (2006).

15. Anitha A, Banerjee M. Arylamine N-acetyltransferase 2 polymorphism in the ethnic populations of South India. *Int. J. Mol. Med.* **11**, 125-131 (2003).

16. Singh N, Dubey S, Chinnaraj S, Golani A, Maitra A. Study of *NAT2* gene polymorphisms in an Indian population: association with plasma isoniazid concentration in a cohort of tuberculosis patients. *Mol. Diagn. Ther.* **13**, 49-58 (2009).

17. Guo WC, Lin GF, Zha YL, Lou KJ, Ma QW, Shen JH. N-Acetyltransferase 2 gene polymorphism in a group of senile dementia patients in Shanghai suburb. *Acta Pharmacol. Sin.* **25**, 1112-1117 (2004).

18. Song DK, Xing DL, Zhang LR, Li ZX, Liu J, Qiao BP. Association of NAT2, GSTM1, GSTT1, CYP2A6, and CYP2A13 gene polymorphisms with susceptibility and clinicopathologic characteristics of bladder cancer in Central China. *Cancer Detect. Prev.* **32**, 416-423 (2009).

19. Tanaka E, Taniguchi A, Urano W, Nakajima H, Matsuda Y, Kitamura Y, Saito M, Yamanaka H, Saito T, Kamatani N. Adverse effects of sulfasalazine in patients with rheumatoid arthritis are associated with diplotype configuration at the N-acetyltransferase 2 gene. *J. Rheumatol.* **29**, 2492-2499 (2002).

20. Deguchi M, Yoshida S, Kennedy S, Ohara N, Motoyama S, Maruo T. Lack of association between endometriosis and N-acetyl transferase 1 (NAT1) and 2 (NAT2) polymorphisms in a Japanese population. *J. Soc. Gynecol. Investig.* **12**, 208-213 (2005).

21. Lee KM, Park SK, Kim SU, Doll MA, Yoo KY, Ahn SH, Noh DY, Hirvonen A, Hein DW, Kang D. N-acetyltransferase (NAT1, NAT2) and glutathione S-transferase (GSTM1, GSTT1) polymorphisms in breast cancer. Cancer Lett. **196**, 179-186 (2003).

22. Lee SY, Lee KA, Ki CS, Kwon OJ, Kim HJ, Chung MP, Suh GY, Kim JW. Complete sequencing of a genetic polymorphism in NAT2 in the Korean population. *Clin. Chem.* **48**, 775-777 (2002).

23. Yuliwulandari R, Sachrowardi Q, Nishida N, Takasu M, Batubara L, Susmiarsih TP, Rochani JT, Wikaningrum R, Miyashita R, Miyagawa T, Sofro AS, Tokunaga K. Polymorphisms of promoter and coding regions of the arylamine N-acetyltransferase 2 (NAT2) gene in the Indonesian population: proposal for a new nomenclature. *J. Hum. Genet.* **53**, 201-209 (2008).

24. Jorge-Nebert LF, Eichelbaum M, Griese EU, Inaba T, Arias TD. Analysis of six SNPs of NAT2 in Ngawbe and Embera Amerindians of Panama and determination of the Embera acetylation phenotype using caffeine. *Pharmacogenetics* **12**, 39-48 (2002).

25. Martinez C, Agundez JA, Olivera M, Llerena A, Ramirez R, Hernandez M, Benitez J. Influence of genetic admixture on polymorphisms of drug-metabolizing enzymes: analyses of mutations on NAT2 and C gamma P2E1 genes in a mixed Hispanic population. *Clin. Pharmacol. Ther.* **63**, 623-628 (1998).

26. Stephens M, Donnelly P. A comparison of bayesian methods for haplotype reconstruction from population genotype data. *Am. J. Hum. Genet.* **73**, 1162-1169 (2003).

27. Excoffier L, Slatkin M. Maximum-likelihood estimation of molecular haplotype frequencies in a diploid population. *Mol. Biol. Evol.* **12**, 921-927 (1995).

28. Barrett JC, Fry B, Maller J, Daly MJ. Haploview: analysis and visualization of LD and haplotype maps. *Bioinformatics* **21**, 263–265 (2005).

29. Clark AG. Inference of haplotypes from PCR-amplified samples of diploid populations. *Mol. Biol. Evol.* **7**, 111-122 (1990).
